# Investigating Word Order Emergence: Constraints From Cognition and Communication

**DOI:** 10.3389/fpsyg.2022.805144

**Published:** 2022-04-22

**Authors:** Marieke Schouwstra, Danielle Naegeli, Simon Kirby

**Affiliations:** ^1^Institute for Logic, Language and Computation (ILLC), University of Amsterdam, Amsterdam, Netherlands; ^2^Department of Communication and Cognition, Tilburg University, Tilburg, Netherlands; ^3^Centre for Language Evolution, University of Edinburgh, Edinburgh, United Kingdom

**Keywords:** silent gesture, word order, reversible events, information theory, verb semantics, interaction

## Abstract

How do cognitive biases and mechanisms from learning and use interact when a system of language conventions emerges? We investigate this question by focusing on how transitive events are conveyed in silent gesture production and interaction. Silent gesture experiments (in which participants improvise to use gesture but no speech) have been used to investigate cognitive biases that shape utterances produced in the absence of a conventional language system. In this mode of communication, participants do not follow the dominant order of their native language (e.g., Subject-Verb-Object), and instead condition the structure on the semantic properties of the events they are conveying. An important source of variability in structure in silent gesture is the property of reversibility. Reversible events typically have two animate participants whose roles can be reversed (girl kicks boy). Without a syntactic/conventional means of conveying who does what to whom, there is inherent unclarity about the agent and patient roles in the event (by contrast, this is less pressing for non-reversible events like girl kicks ball). In experiment 1 we test a novel, fine-grained analysis of reversibility. Presenting a silent gesture production experiment, we show that the variability in word order depends on two factors (properties of the verb and properties of the direct object) that together determine how reversible an event is. We relate our experimental results to principles from information theory, showing that our data support the “noisy channel” account of constituent order. In experiment 2, we focus on the influence of interaction on word order variability for reversible and non-reversible events. We show that when participants use silent gesture for communicative interaction, they become more consistent in their usage of word order over time, however, this pattern less pronounced for events that are classified as strongly non-reversible. We conclude that full consistency in word order is theoretically a good strategy, but word order use in practice is a more complex phenomenon.

## Introduction

Many languages in the world have a dominant pattern for ordering the Subject, Object, and Verb in sentences, and of the dominant orders of existing languages, SOV and SVO are the most frequent (Dryer, 2005; Napoli and Sutton-Spence, 2014). One of the big questions in linguistics is: why do languages of the world look the way they do? In trying to map out how conventions for basic word order arose, one of the methodologies that have been applied is silent gesture (Goldin-Meadow et al., 1996, 2008; Gershkoff-Stowe and Goldin-Meadow, 2002). In silent gesture experiments, participants are asked to convey information using only gesture and no speech. When they do this, participants do not tend to simply follow the dominant order of their native language; instead, participants from various language backgrounds show striking similarities in the ways in which they structure information, influenced by the semantic properties of the message to be conveyed (Gibson et al., 2013b; Hall et al., 2013; Schouwstra and de Swart, 2014; Meir et al., 2017; Kirton et al., 2021). In this way, silent gesture experiments have been a rich source of evidence about the origins of word order conventions in human language. The method allows us to investigate how emerging languages are shaped and, eventually, why the languages we know today look the way they do. Note that apart from word order, other ways of conveying who did what to whom, such as morphological strategies in free word order languages (see Levshina, 2021, for a recent discussion of trade-offs between morphological marking and word order), and spatial strategies in sign languages, are possible too, and it is also possible to study these using silent gesture (Motamedi et al., 2021a). We will discuss other ways to mark semantic roles in the general discussion, but for now our main focus is word order.

In making the step from silent gesture to general conclusions about language structure, different potential mechanisms have been proposed, relying on cognitive biases in individuals (Gibson et al., 2013b), communicative principles (Meir et al., 2010), or biases coming directly from (gestural) language production (Hall et al., 2013, 2015). How these accounts relate to each other, or which of them is the right one, is still an open question. A big discussion has revolved around *reversible* vs. *non-reversible events*. In this paper, we will propose a new, more detailed, analysis of reversibility and present two experiments (one silent gesture production and one silent gesture interaction experiment) that provide new evidence about their role in emerging word order conventions.

### Reversibility and Word Order: Rooted in Production, Cognition, or Communication?

Reversible events are generally described as transitive events in which the agent and patient are both human, and therefore plausible as agents. In these events, the roles of agent and patient can plausibly be switched; for example, *girl kicks boy*. Non-reversible events are those events for which the roles cannot be plausibly switched; for example, *girl kicks ball* (adopting terminology from Hall et al., 2013; Futrell et al., 2015).

For non-reversible events, the preferred order in silent gesture is SOV, irrespective of the language background of the participants (e.g., Goldin-Meadow et al., 2008). Given that SOV order is the most frequent dominant order in the languages of the world, and often observed as the dominant order in emerging sign languages, this order is often considered the default basic word order (Newmeyer, 2000). For reversible events, on the other hand, participants, again from different language backgrounds, deviate from SOV order, most frequently to SVO order (a.o., Gibson et al., 2013b; Hall et al., 2013; Futrell et al., 2015; Meir et al., 2017). A central question, therefore, is where these ordering preferences come from.

A dominant account of basic word order in human language is the *noisy channel account*, proposed by Gibson et al. (2013b) and supported and expanded in Futrell et al. (2015). In this account, as in others, the SOV preference for non-reversible transitive events is ascribed to a cognitive bias in favor of presenting Subject information first, and relational information last. To explain the shift away from SOV to SVO the noisy channel account appeals to information theory and takes as a central observation that communication is a noisy phenomenon, and communicators strive to minimize the potential for errors on the side of receivers. If part of an SOV ordered message (S or O) were to be lost due to noise, and the remaining element can plausibly be the Subject or Object, then it is impossible to deduce whether the remaining information is a Subject-Verb or an Object-Verb pair. Verb medial orders, by contrast, are more robust to noise, because even when information about one of the participants is canceled out, an interlocutor can tell solely from the position with respect to the verb, whether the remaining information concerns the Subject or the Object.

To summarize, the noisy channel account takes as a starting point two cognitive biases that drive word order preferences initially: a Subject-initial and a (weaker) Verb-final bias. It explains the usage of SVO for reversible events by appealing to a communicative principle, observing that reversible events are especially sensitive to noise, and that SVO order is more robust (Futrell et al., 2015).

The noisy channel account has been challenged. The *role conflict account*, put forward by Hall et al. (2013), appeals to principles purely connected to language production in the gestural domain. The authors postulate that for reversible events, producers of silent gesture prefer to move away from SOV order, because that order would force them to first embody the role of the subject, then that of the direct object, and then, implicitly, the role of the subject again (in conveying information about the action and using “body as subject”; Meir et al., 2007).

The two accounts emphasize different processes driving word order conventions. The role conflict account is modality-specific and production-specific: It emphasizes the processes that take place when individuals *produce* improvised, gestural descriptions. The noisy channel account, by contrast, is rooted in general principles of cognition and communication. The two accounts make different predictions about potential other orders (apart from SOV and SVO) observed in silent gesture experiments, but also about the generality of the SOV/SVO pattern. The role conflict account would predict that the spoken modality does not face the same problems for reversible events, and the word order pattern described here is thus expected to be a property exclusively of language in the visual manual domain. The noisy channel account, on the other hand, assumes that the pattern is driven by general cognitive/communicative biases, and would predict it to be a property of language in general, independently of the modality.

Several lab studies addressed the modality specificity of the reversibility word order pattern directly, by attempting to replicate the SOV/SVO alternation in a modality different from the visual manual (as this would be strong evidence in favor of a general cognitive, rather than a modality specific, account; Struhl et al., 2017; Kirton, 2021). So far, these studies have not been able to replicate the effect in a different modality, although, Fedzechkina et al. (2012) did show that learners of a spoken artificial language avoid SOV order for reversible events when this language did not have case marking. However, the study did not replicate the SVO preference (due to the fact that this was a learning experiment, and participants were trained on V-final languages). To date, there is no clear replication of the SOV/SVO pattern for reversibility in the non-manual domain.

One other source to turn to, looking for evidence for the nature of word order constraints are new sign languages. For some of these languages, there is rich documentation of the linguistic patterns present in different stages of emergence, including word order. In a number of emerging sign languages (e.g., Senghas et al., 1997; Meir et al., 2010; see Kirton, 2021, for an overview), so-called *paired verb constructions* (Flaherty, 2014) have been observed, in which an event is described using two consecutive verb-argument pairs, one that contains actor information, and one that contains patient information. An example is MAN TICKLE; WOMAN GET-TICKLED to convey “man tickles woman.” In the emerging sign languages mentioned here, the paired verb construction is more frequent for reversible than for non-reversible events. The dispreference in signers of emerging sign languages to combine verbs with more than one argument, particularly for reversible events, might be production-specific in nature, but it might also be communicative. The paired verb construction solves potential role conflict problems in production in the visual-gestural domain, but it also solves the potential communicative problem of the inherent unclarity about who does what to whom. It does that, not in the same way as the noisy channel account proposes, but in a way that still makes the utterance more resistant to noise.

All in all, data from existing languages presents potential support for the role conflict account as well as the noisy channel account of word order, and in the current paper, we will focus on finding laboratory evidence (but we recognize that it will be interesting to test our lab results, and the predictions they generate, against natural data from existing languages). We will address two topics that help gain insight into the emergence of word order conventions. The first concerns the definition of reversibility, and the second consistency in gesture production by lab participants. We will show that we can use these perspectives to gain new insights into the driving forces of word order conventions.

### Reversibility as a Composite Concept

In the literature, reversibility has always been defined in terms of the animacy of the agent and patient. Most studies have focused on animate-animate reversible events (*girl kicks boy*), except for Kocab et al. (2018), who also focus on inanimate-inanimate reversible events (*car hits truck*). None of the papers, however, focuses directly on the properties of the *action*. Here we explain that the properties of the verb *are* relevant in the discussion about word order biases. We hypothesize that reversibility is, contrary to what has been assumed so far, a **composite** phenomenon; the properties of the patient, as well as the properties of the action together determine the extent to which an event is reversible.

Central to our proposed analysis is the idea that the meaning of a complex expression is determined by the meanings of the parts, plus the way in which they are put together. This is the property of compositionality, and we propose that the extent to which an event is reversible is determined compositionally, by the properties of the participants in an event, but also the properties of the action. To see why properties of an action play a role in the extent to which an event is reversible, let’s first focus on different verbs, describing different kinds of actions, and the way in which they behave with respect to the arguments they tend to take.

Consider the verbs “to eat” and “to kick.” Both verbs are transitive, but we do not see them combined with the same kind of arguments. Focusing on the patient role, we can see that the verb *kick* can combine with a variety of nouns. *Man, car, box, banana, cow, biscuit, tree, window* are all felicitous direct object arguments for *kick.* The verb *eat*, however, behaves differently. Some of the verb-direct object combinations, like *eat a box, eat a window*, are not very likely to occur; these combinations lead to a semantic clash. In other words, verbs behave differently with respect to the kinds of arguments they combine with.

This idea is not new; in fact, the term *selectional restrictions* was introduced into linguistic theory many decades ago by Katz and Fodor (1963), and also discussed by Chomsky (1965), as lexicon-internal constraints that verbs place on their arguments. Under this interpretation, selectional restrictions are different from world knowledge, and strictly part of linguistic systems. Others, like Johnson-Laird (1983, 1987), presented arguments in favor of a view that selectional restrictions are the result of an inferential process, based on world knowledge, a graded notion, and essentially rooted in properties of the world. Consistently with the latter, we propose that something like selectional restrictions can play a role in the way people convey information, even when people do not rely on existing language conventions (like in silent gesture).^1^

Of the two example verbs described above, *kick* is much more likely to occur in reversible events than *eat.* However, *kick* is not necessarily reversible: if it is combined with an animate agent and an inanimate patient, the resulting event (e.g., *boy kicks watermelon*) is non-reversible. By contrast, a verb like *eat* is non-reversible, but the action of eating will be very unlikely to occur in a reversible event. In other words, non-reversible events, which are treated in the literature as an unanalyzed class of events, come in different flavors with respect to their potential for reversibility.

To sum up, there are non-reversible events (like *boy kicks watermelon*) that can be made reversible by changing the participants in the event. We will call these **weakly non-reversible events**. Some actions, by contrast (e.g., *boy eats watermelon*) are unlikely to ever occur in a reversible event, because of the selectional restrictions of the verb on its arguments. These actions make an event non-reversible in a stronger sense: we cannot make these events reversible by changing one of the participants. We will call these **strongly non-reversible events**. Due to their differences in patient animacy and verb properties, the three event types differ crucially in their potential for role confusion of the event participants, which is illustrated in Table 1.

**TABLE 1:** Event types and their properties.

Event type	Properties of patient	Property of verb (action)	Example	Potential role confusion
Reversible	Animate	Reversible	*Boy punches man*	High: both participants can plausibly take both roles
Weakly non-reversible	Inanimate	Reversible	Boy punches watermelon	Medium: Agent could plausibly be patient, but patient unlikely to be agent
Strongly non-reversible	Inanimate	Non-reversible	Boy eats watermelon	Low: Agent unlikely to be patient, patient unlikely to be agent

This makes reversibility a composite concept: the properties of multiple relevant elements of an event together (rather than just the properties of the patient) determine the extent to which the event is reversible. In Experiment 1 we test the hypothesis that the extent to which an event is (non) reversible influences the word order preferences participants have. We hypothesize that if both patient properties and verb properties are important for reversibility, a difference in strength of the SOV preference is not only visible between reversible and non-reversible events, but also between weakly non-reversible and strongly non-reversible (such that SOV is more strongly preferred for strongly non-reversible events).

### Word Order Consistency

An obvious and effective strategy to remedy the inherent ambiguity that comes with the description of reversible events is to have a word order convention. When there is a convention for word order (e.g., to use SVO consistently), having animate agent and patient will not give rise to ambiguity. One could imagine that having to communicate about reversible events could trigger consistent word order use. The notion of consistency is based on communication, like the noisy channel account, but is more general, in that it does not prescribe a specific word order: any order could work; the strategy relies on the simple fact that forming a convention could decrease the uncertainty about who does what to whom. If we would find evidence for this strategy, it would mean that communicative principles do play a role in the formation of word order conventions (contrasting with the view, e.g., in Langus and Nespor, 2010, that word order preferences essentially stem from cognitive processes).

We are not the first to consider word order consistency in combination with reversibility. Hall et al. (2013) investigated whether the presence of reversible events influences the SOV preference for subsequent non-reversible events in the same silent gesture experiment. Finding this kind of evidence, the authors claim, would suggest an important role for reversible events in pushing SVO word order to become dominant in languages. They ran a silent gesture production study with three conditions: one that had all reversible events first, one that had all reversible events last, and one that had the two event types interleaved. They observed that the SOV preference differed in the expected direction, but could not statistically confirm this observation.

To see how the types of reversibility defined here behave with respect to word order consistency, we incorporated order of presentation into our experiment design. Consistency in word order is an intuitively appealing strategy to eliminate the inherent ambiguity that comes with the description of reversible events, and our new analysis of reversibility allows us to investigate this in more detail. We hypothesize that if encountering reversible events pushes a bias for word order consistency, this will lead to higher proportions of SVO for non-reversible events when reversible events are presented first (as compared to when reversible events are presented last).

## Experiment 1: Reversibility in Silent Gesture Production

To test the two hypotheses formulated above, we ran a silent gesture production study that investigates the nature of reversibility and the issue of word order regularity. The preregistration for this study can be found on https://osf.io/pnk9s. Our experiment has two conditions: in the reversible-first condition, participants see a series of only reversible events, followed by a series of (weakly and strongly) non-reversible events; in the reversible-last condition, participants first see a series of non-reversible events, followed by a series of reversible events. Concerning the two types of non-reversible events, and given the first hypothesis above (at the end of section “Method”), we predict a word order difference, such that weakly non-reversible events behave more like reversible events (i.e., elicit fewer SOV strings) than the strongly non-reversible events. Concerning word order consistency, following the second hypothesis above (at the end of section “Word Order Consistency”), we predict lower overall proportions of SOV for non-reversible events when participants describe reversible events first than when they describe reversible events after the non-reversible events.

### Method

**FIGURE 1:**
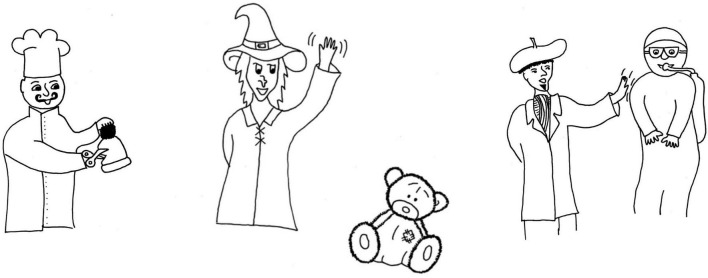
Examples of events used in Experiment 1. From left to right: “Chef cuts hat” (strongly non-reversible), “Witch waves at teddy bear” (weakly non-reversible), and “Artist taps scuba diver” (reversible).

Thirty-six line drawings were created for the experiment, twelve in each of the categories reversible, weakly non-reversible, and strongly non-reversible. See Figure 1 for an example image of each type. Even though the actions used in the weakly non-reversible events were in principle suitable to be used in reversible events too, we used unique actions in each of the categories, to avoid potential effects of repetition. The full set of images can be found on https://osf.io/2pr48/.

**FIGURE 2:**
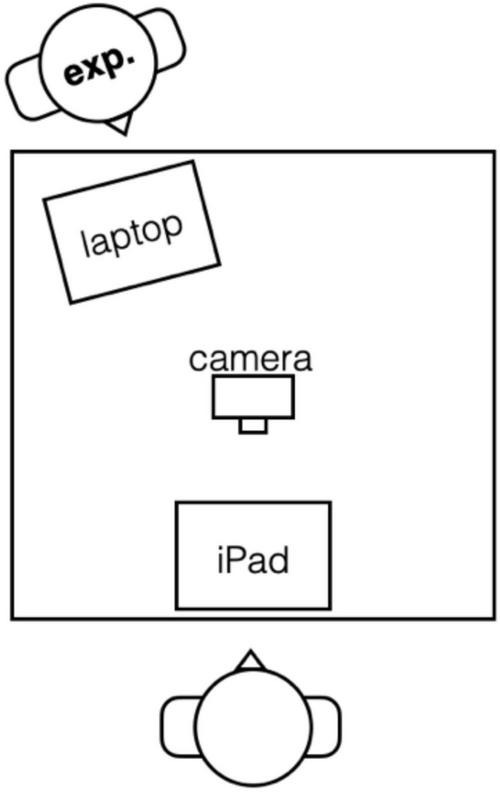
Experiment setup: the participant sat across the table from the experimenter, who showed the images on an external screen placed in front of the participant.

Stimuli were presented on an iPad that was placed on a table in front of the participant, displaying the images through dedicated Psychopy software (Peirce et al., 2019). Participants were filmed using a Logitech webcam connected to the experimenter’s laptop (see Figure 2).

There were two conditions, each consisting of two stages. In the reversible-first condition, only reversible events were presented in the first stage, and only (weakly or strongly) non-reversible events in the second stage; in the reversible-last condition, this order was reversed. Participants were each assigned to one of the two conditions at random. Thirty participants took part in the study (15 in each condition).^2^ The participants were recruited from a University of Edinburgh website used for advertising casual employment. They reported having no experience with any signed language and were all first language speakers of English.

Both experiments reported in this paper were approved by the Philosophy Psychology and Language Sciences Ethics Committee at the University of Edinburgh. All participants gave consent prior to beginning their session. This included permission to share videos or images from their sessions for research purposes as well as with the general public. Four participants agreed to include their video recordings for analysis, but not for publication to the general public.

The participant was seated across the table from the experimenter. Stimuli were presented on an external screen, and the session was video recorded. After signing the consent form, the participant was instructed that they would see line drawings on the screen, and it was their task to convey what they saw, using only gesture, and no speech. After each trial, the experimenter moved the experiment on to the next trial. Within each stage of the experiment, stimuli pictures were presented in random order (a different order for each participant). In each trial, a stimulus picture was presented either in its original orientation or as a mirror image, to avoid left-to-right effects of Agent and Patient.

Before starting the actual experiment, the participant saw two practice trials. These were from the event category of the first stage of their session (depending on the condition). The images used as practice trials were taken from a separate set and contained no elements (Agents, Patients, Actions) that occurred in the actual experiment. During the practice trials, participants did not get specific feedback on the contents of what they conveyed, but they were encouraged to convey more detail if they only provided one or two of the target Agent, Patient, and Action.

The video recordings were coded for the word orders used by participants. Subsequently, the coded word orders were categorized as SVO, SOV, or Other/NA for this analysis. The latter included any orders different from SVO and SOV (this included incomplete or repetitive gesture strings). Per condition (reversible-first/reversible-last) one participant’s recordings (selected at random) were also coded by an independent second coder. The proportional agreement between the first and second coder was at 0.902. This corresponds to a Kappa value of 0.83, which is interpreted as almost perfect agreement. [Note that McHugh (2012) disagrees with the interpretation of the Kappa value used here, and proposes to use it only with Kappa > 0.9. However, following the logic of that paper, using the raw proportion agreement is a better guide, which, at > 0.9, is classified as “very high”].

### Analysis and Results

**FIGURE 3:**
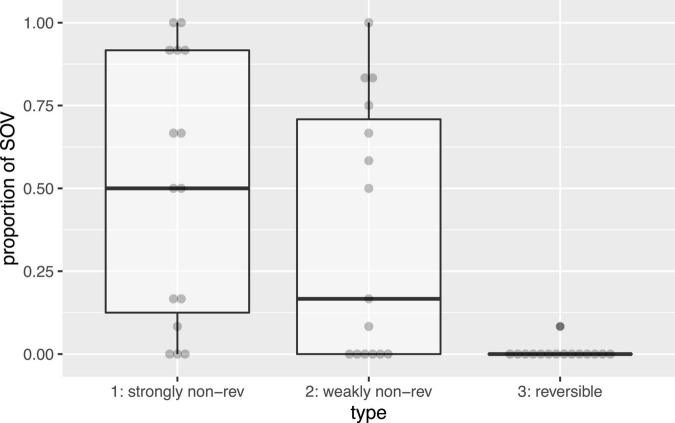
Proportion of SOV responses by type of event. Gray dots represent participants. Strongly non-reversible events elicited more SOV responses than weakly non-reversible events. (Reversible events are displayed for reference, but were not included in the analysis. See data repository for a full analysis).

In accordance with the preregistered analysis plan, our analysis focused on levels of SOV order, and assessed effects of condition and event type. To find out if weakly non-reversible events behaved differently from strongly non-reversible events, we focused on the non-reversible events in the reversible-last condition and compared the proportion of SOV strings for strongly non-reversibles and weakly non-reversibles, respectively. We ran a binomial mixed-effects regression that modeled SOV, taking item-type (weakly vs. strongly non-reversible) as fixed effect, by-participant random intercepts and slopes for item-type, and by-item random intercepts.^3^ The model reveals a significant main effect of non-reversibility-type (beta = 1.60, SE = 0.58, *p* = 0.006), allowing us to conclude that the distinction between weakly and strongly non-reversible events is reflected in the word orders used for these events in silent gesture. Figure 3 shows this preference: strongly non-reversible events elicit higher proportions of SOV order than weakly non-reversible events.

**FIGURE 4:**
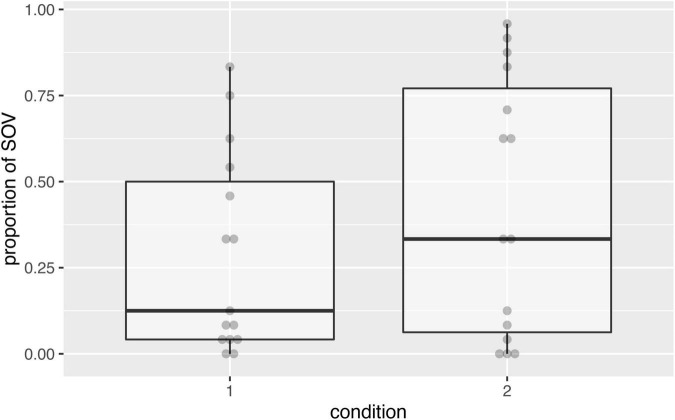
Graph showing the proportion of SOV responses to both types of non-reversible events by condition. Gray dots represent means per participant. In condition 1 (reversible events first), the median level of SOV was lower than that in condition 2 (reversible events last) as predicted by the hypothesis that word-order consistency is a strategy to deal with potential ambiguity. However, the difference between the conditions was not significant.

To assess if the order of presentation matters for the proportion of SOV used in (both types of) non-reversible events, we compared the usage of that order in the two conditions, using a binomial mixed-effects regression that modeled SOV, taking condition as a fixed effect, by-participant random intercepts, and by-item random intercepts (including slopes for condition resulted in a singular fit). The model did not show a significant main effect of condition (beta = 0.92, SE = 0.91, *p* = 0.31). Figure 4 shows that the mean level of SOV is higher when non-reversible events are presented first. However, due to the lack of significance (and consistently with Hall et al., 2013), we cannot conclude that the order of presentation of reversible and non-reversible events influences the level of SOV preference for non-reversible events.

### Discussion

Our experiment confirmed that word order preferences based on reversibility are more complex than previously thought: events that are strongly non-reversible (with the non-reversibility residing in the patient properties as well as in the properties of the action) led to higher proportions of SOV in silent gesture than events that are only weakly non-reversible (with the action being potentially reversible). Relating these findings to the two accounts discussed above, we can conclude that the role conflict account cannot be the full explanation of word order variability connected to reversibility: both types of non-reversible events used in the current experiment have the same assignment of participants: a human agent and an inanimate patient. In both cases, no role conflicts are expected, and the role conflict account cannot explain the difference in word order here. The consequences of our findings for the noisy channel account are harder to assess; it is dependent on whether weakly non-reversible events are more susceptible to noise or not. We will discuss this in detail in the general discussion.

Our experiment failed to find evidence that word order preferences are influenced by the order in which the stimuli are presented (whether our participants started with a series of reversible trials or with non-reversible trials). In this respect, our results are similar to Hall et al. (2013), and given that this is a null result, we cannot connect strong conclusions to it. However, we tested only single participants, and the experiment lasted for a relatively short duration, so perhaps for consistency to become a relevant pressure, we might need a more communicative setting.

## Experiment 2: Reversibility in Silent Gesture Interaction

**FIGURE 5:**
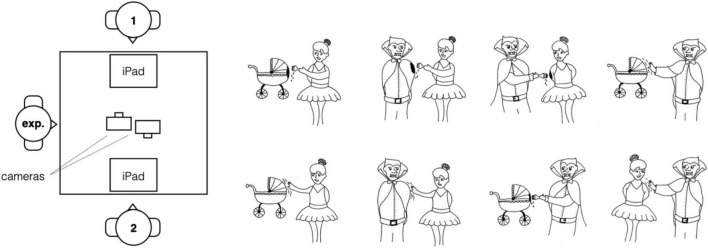
Left: setup overview of Experiment 2. Right: example of stimuli screen with 8 events as shown to matcher.

The preregistration of this experiment can be found on https://osf.io/unw98. Eight sets of stimuli pictures were created, each set consisting of eight line drawings of transitive events. All sets contain all eight possible combinations of one of two actions, one of two agents, and one of two patients. Two sets contained only strongly non-reversible events, and the other six contained combinations of weakly non-reversible and reversible events. Figure 5 shows an example of one of the latter. In the reversible events, one character has the role of agent and the other patient. All eight sets of stimuli images can be found on https://osf.io/fk6yq/.

Stimuli pictures were shown to the participants on iPads through networked software running from a laptop server. The software and details of the procedure were very similar to that of experiment 2 in Schouwstra et al. (2020). Stimuli pictures were randomly shown as the original version or mirrored to avoid a potential left-to-right bias due to the order of the elements in the picture. Two Logitech cameras were used to film the participants. The cameras were connected to laptops operated by the experimenter.

Twenty four participants took part in the experiment, in pairs. The participants were recruited individually, via a University of Edinburgh website used for advertising casual employment (MyCareerHub).^4^ All participants were native speakers of English and had no previous experience with sign languages. Furthermore, all participants consented to being video-recorded and having their videos analyzed ahead of the experiment, but three participants opted out of giving permission to use their videos publicly.

Pairs of participants engaged in a silent gesture interaction experiment seated opposite each other, both being video recorded independently. The experimenter was present in the room and seated to the side (see Figure 5).

In a director-matcher task, the participant in the director role was asked to convey information about a stimuli picture to the other participant. The other participant’s task (as the matcher) was to pick the target stimulus out of an array of eight pictures consisting of all the pictures belonging to the same set (see Figure 5 for an example). The matcher set always consisted of all 8 combinations of 2 agents, 2 patients, and 2 actions, to ensure that information about all elements was needed for the matcher to make their choice.

Participants switched roles after each trial and completed six rounds with 32 trials per round (with one of the participants always starting in the director role). Because there were 64 stimuli pictures in total, participants did not see all of these in the first round (where they only saw 32). In subsequent rounds, 16 new pictures were added to replace 16 already-seen pictures, such that after four rounds each picture was described at least once. We made sure that both participants described equal shares of reversible and (strongly/weakly) non-reversible events.

After each trial both participants received full feedback: the director received information about whether the matcher’s response was correct, and if incorrect, which image was chosen. The matcher received information about whether the response was correct, and in incorrect, what the correct image was. To make the experiment more game-like and make participants more engaged, participants were told at the end of each round whether they completed it faster than the previous round. If they achieved this, the experimenter handed out wrapped sweets as rewards. Furthermore, the fastest dyad overall was awarded an extra financial incentive (£5) once all the experiments were conducted.

Of 2,304 trials in total, 3 were skipped without the participant in the director role producing any gestures, because of an error in the software. The remaining 2,301 trials were coded for word order, and, for the purpose of statistical analysis, classified into three categories: SVO, SOV, and Other. The recordings of two dyads were randomly chosen as a sample and then coded again by an independent second coder. The proportional agreement between the first and second coder was 0.97 (this corresponds to a kappa value of 0.89, which is classified as almost perfect agreement).

### Analysis and Results

To assess whether participants became more consistent in their word order use over time (and in accordance with the pre-registered analysis plan), we looked at entropy measures for each pair of participants, and how this developed over time. This was done by calculating Shannon’s entropy for the utterances produced by each dyad, for each round, for the proportions of SOV, SVO, and other word orders. When entropy is calculated for a series of utterances, usage of different word orders in equal proportions results in higher entropy (e.g., 1.58 bits for when three word orders are used in equal proportions), and usage of the same word order for every utterance results in an entropy of 0 bits. Entropy is used in a number of studies to investigate the rate of regularization in learning studies (e.g., Ferdinand et al., 2019); here we use it to keep track of the rate of regularization over time, by calculating the entropy for each round.

**FIGURE 6:**
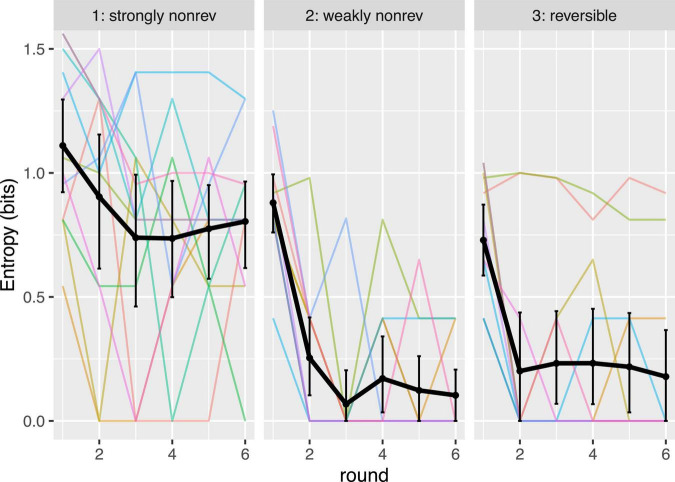
Entropy (bits) by round, for each of the three event types. Black lines represent means, and colored lines represent dyads. Error bars represent bootstrapped 95% confidence intervals. We can observe a drop in entropy over time, on average, supporting the hypothesis that over time, participants become more consistent in their word order use. On average, entropy stays higher for strongly non-reversible events.

To assess if entropy went down over the course of the experiment, we ran a linear mixed-effects model, modeling entropy as the dependent variable, round (log transformed and centered), and event type (simple coded, with strongly non-reversible events as the reference level, and reflecting the grand mean in the intercept) as the independent variables, and including by-participant-pair random intercepts and slopes for round (including event type in the random effect structure resulted in singular fits).^5^ The model reveals a main effect of round on entropy (beta = −0.28, SE = 0.04, *p* < 0.001), confirming that over time dyads regularize their output, following a logarithmic trend (see Figure 6). When event types were compared, a significant effect was found for strongly non-reversible events, both when these were compared to weakly non-reversible events (beta = −0.60, SE = 0.06, *p* < 0.001), and to reversible events (beta = −0.55, SE = 0.06, *p* < 0.001). Focusing on the interactions, there is a significant interaction between round and event type, where strongly and weakly non-reversible events are compared (beta = −0.21, SE = −0.09, *p* = 0.02), but no significant interaction when strongly non-reversible events are compared with reversible events (beta = −0.07, SE = 0.09, *p* = 0.41).

**FIGURE 7:**
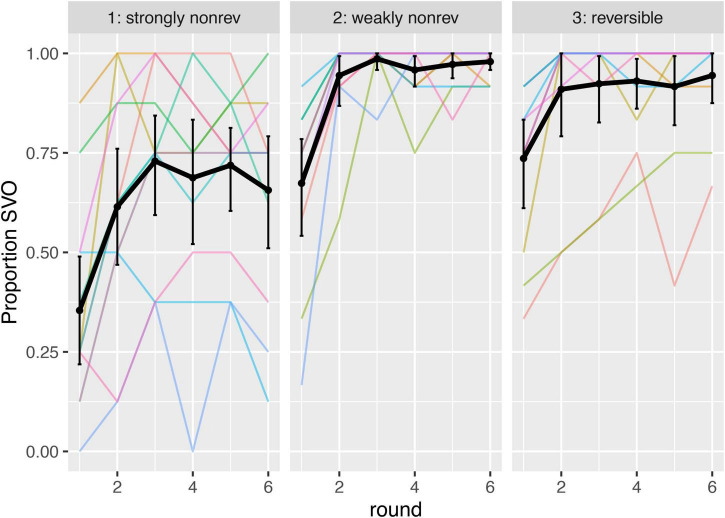
Proportion SVO order by round, for each of the three event types. Black line represents mean, error bars 95% CIs, and colored lines dyads.

To get an idea of how the overall drop in entropy was realized, we looked at the word orders produced in the experiment. Of the 2,301 coded trials, 1,927 were SVO, 119 were SOV, and the remaining 255 were other orders (SVOV was the most frequent of these, with 55 trials; an example is “pirate punch balloon punch.”) Over time, SVO became the most frequently used order by far. This is visualized in Figure 7.

**FIGURE 8:**
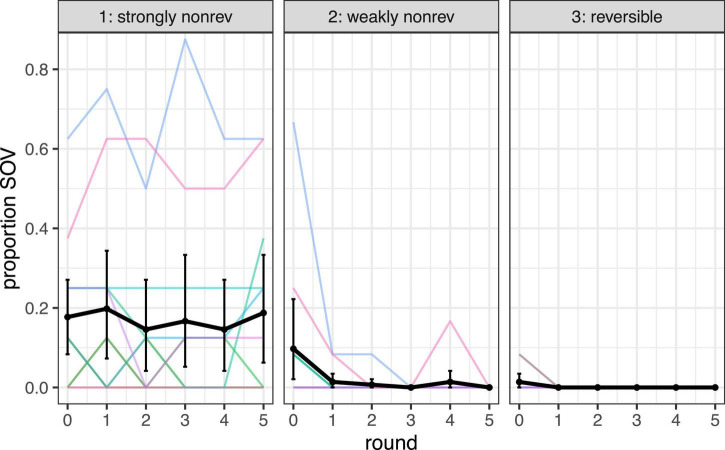
Proportion SOV by round, for strongly non-reversible events (left), weakly non-reversible events (middle), and reversible events (right). Black lines indicate means, error bars are 95% CI’s, and colored lines represent dyads. In the analysis we compared the two non-reversible event types; the reversible events are included for reference.

To get an idea of whether the increase in SVO order was realized by moving away from SOV specifically (or rather by no longer using other word orders), we focused on the proportion SOV, and how it developed over time. Figure 8 shows this, splitted out per event type. From the graph, a few things stand out. First of all, there is a difference in SOV levels: strongly non-reversible events seem to have elicited more SOV. Further, the level of SOV does not seem to decrease as much over time as entropy. Finally, two of the participant pairs produced much more SOV than the others. In what follows, we will first discuss the preregistered model, followed by a model that excludes the data from these two outlier participant pairs.

Our statistical analysis focused on the two non-reversible event types, following the preregistered model. In a logistic mixed effects regression, we predicted SOV (a binary variable, with value TRUE if the string was SOV, and FALSE if it was not), taking round (centered) and event type (sum coded) as predictors, and by-participant-pair random intercepts and slopes (plus their interaction), as well as by-item random intercepts. The model revealed a significant main effect for event type (beta = −4.41, SE = 0.73, *p* < 0.001), showing that overall, SOV was used significantly less often for weakly non-reversible events than for strongly non-reversible events. Further, the model revealed a main effect of round (beta = −3.36, SE = 0.89, *p* < 0.001), showing that the proportion of SOV decreased over time. Finally, there was a significant interaction between round and event type (beta = −5.71, SE = 1.45, *p* < 0.001), confirming that SOV decreased more strongly for the weakly non-reversible events than for the strongly non-reversible events.

When we re-ran our statistical analysis with the two outlier participant pairs excluded, no inference could be made on the basis of the outcome, because of a data separation issue (almost all occurrences of SOV were in the strongly non-reversible category). The model and its outcome can be found on https://osf.io/fk6yq/?view_only=000d1f209ca449c2ba0ce48b59a91d9d. All in all, we could not get a clear enough picture of what happened to SOV word order over the course of the experiment and in the different event types, and a larger data set would be necessary to assess whether the two removed participant pairs were a rarity, or simply a somewhat less frequent strategy.

Note that the preregistration mentions two further measures, of lineage specificity, and structural priming. We decided not to include these in our analysis, because each topic connects to an extended literature, which diverts too much from the current topic of the paper.

### Discussion

Our findings confirm that, first of all, participants become more regular in their usage of word orders over the course of the experiment; this was shown by a significant decrease in entropy over time. At the same time, there is a difference in entropy between the two types of non-reversible events: entropy remains higher for strongly reversible events than for weakly reversible events. It should be noted at the same time that the main decrease in entropy took place in the first few rounds of the experiment, and remained relatively stable in later rounds; pairs of participants did not reach fully regular word order usage, particularly for strongly non-reversible events.

The fact that the dominant word order is SVO in all of our participant pairs could be partly a consequence of the fact that all our participants were speakers of English, but it is also consistent with the observation that SVO is a more stable word order for reversible events than SOV.

## General Discussion

What are the driving forces behind basic word order conventions? In the literature, different accounts have been proposed, focusing on cognitive, communicative, and production-specific biases. We have added to the ongoing discussion by focusing on (1) the definition of reversibility, and (2) the process of regularization in communicative interaction.

### Redefining Reversibility

We have refined the concept of reversibility, by proposing that the extent to which an event is reversible is determined by not only the animacy properties of the patient but also the properties of the action. Experiment 1 confirmed that in silent gesture production, participants use different orders for events that only differ in the properties of the action: for events that have an inanimate patient and contain an action that is potentially reversible (like “girl punches watermelon”), participants’ gestural descriptions show lower proportions of SOV than events that have inanimate patients but actions that are less suitable for role reversal (like “girl eats watermelon.”) The latter behave much more like reversible events and elicit higher proportions of SVO.

Above, we already pointed out that this finding has consequences for the role conflict account of reversibility. The pattern we observed cannot be explained with appeal to purely gesture-production specific processes: after all, neither of the two event types we contrasted give rise to role conflict in gesture production. The question remains, then, if the pattern can be explained by the noisy channel account. The answer to this question depends on whether we think there is a difference in susceptibility to noise between the two kinds of non-reversible events. For reference, let us first look in detail at the susceptibility to noise of *reversible* events. Consider the following example:

(1) GIRL BOY KICK
a. GIRL KICK
b. BOY KICK

The sentence in (1) represents an SOV description of a reversible event, and the expressions in (1a) and (1b) represent cases in which either the Subject or the Object has been deleted (we are not considering the case of the verb being deleted). The argument is (Futrell et al., 2015) that when SOV order is used, and noise cancels out one of the arguments of the verb, it is not possible to infer the role of the remaining argument: in (1a) we do not know if the girl is doing the kicking or being kicked, and similar for the boy in (1b). Now, is an event like “girl kicks watermelon” more sensitive to noise than an event like “girl eats watermelon”? Let’s consider the possibilities:

(2) GIRL WATERMELON KICK
a. GIRL KICK
b. WATERMELON KICK

(3) GIRL WATERMELON EAT
a. GIRL EAT
b. WATERMELON EAT

The two sentences in 2 and 3 represent SOV-ordered descriptions of events, and the a and b sentences represent cases where one of the nouns has been deleted. Now, can we infer who is doing what after observing these noise-affected sentences? For both b-sentences, this is not a problem: WATERMELON is very likely to take the patient role, whatever the action is. For the a-sentences, however, a difference arises: GIRL is not a very likely patient for the action EAT, but GIRL *is* a potential patient for the action KICK. In other words, there is a difference between these events, and strongly non-reversible events are more robust to noise than weakly non-reversible events. Because of this difference, the noisy channel account predicts a difference in word order preference for the two events (even though this has never been described explicitly in the literature): weakly non-reversible events behave more like reversible events. Our experiments confirm this pattern.

The differences between the event types we have regarded so far revolve around the inherent ambiguity of the roles in an event description. For reversible events, this ambiguity is obvious: from a combination of a verb and two human arguments, we cannot tell without having syntactic information, who is doing what to whom. For a combination of a verb plus one human and one inanimate argument, the roles are inherently clear as long as all the information is there, but noise can potentially lead to ambiguity (as in the GIRL KICK example above). Only for strongly non-reversible events, this ambiguity is very unlikely to occur (for GIRL EAT, a patient role for GIRL is just not very likely).

Our results confirm that the SOV preference in strongly non-reversible events is greater than in weakly non-reversible events, and this pattern is well explained by the noisy channel account of word order. In other words, our data is in accordance with the general idea that human cognition is well set up for dealing with communication that is potentially faulty or incomplete (Levy et al., 2009; Gibson et al., 2013a).

We should be aware, though, that in this experiment we have looked at word order in isolation, whereas, like it was mentioned in the introduction, there are other potential ways of conveying who did what to whom. In spoken language, this can be done with morphological means, and in sign languages, spatial strategies are often used to disambiguate information. Future experimental work can extend the word-order-only approach to a more complete picture of linguistic conventions.

### Word Order Regularization in Communicative Interaction

In Experiment 2 we carried out an interactive experiment to further investigate how word order biases are affected in actual communication. We showed that over time, communicators decrease the variation in their silent gesture output, arriving at a more regular word order regime in which SVO is the dominant order. This pattern is consistent with the intuition that having a word order convention is helpful when communicators are conveying information about events where the roles are not inherently clear, and with the fact that SVO more reliably conveys information about reversible events. SVO was also the word order of the native language of all participants, so we should consider the possibility that our participants simply fell back to the order of English, also because they knew that they were communicating with another English speaker. This is possible of course. But if we regard our results in the light of previous results, it does not seem entirely straightforward.

In earlier work in which participants communicate using silent gesture, other semantic distinctions were investigated: manipulation vs. creation events (Christensen et al., 2016), and extensional vs. intensional events (Schouwstra et al., 2020). In these experiments, like in the present one, participants became less variable in their word order usage over time when they interacted using silent gesture. SVO word order is less prominent in these earlier studies, though, with Christensen et al. (2016) observing that SOV remains the dominant order (even though their participants are speakers of Danish, an SVO dominant language), and Schouwstra et al. (2020) observing that the word order preference is modulated by event frequency. In other words, it is not necessarily true that in a silent gesture communication task, participants simply fall back to their native language. The high proportions of SVO order might be meaningful in this sense. Eventually, the only way to reach conclusive evidence about this is to repeat the experiment with speakers of a language that is not SVO-dominant.

In our experiment, we saw a decrease in entropy over time, but at the same time, none of the dyads reached full word order consistency. Instead, entropy remained quite stable in the second half of the experiment. Our lab observations are consistent with word order data on reversible vs. non-reversible events from existing languages in that respect, but then we should ask the question: if having a consistent word order helps in reliably conveying information about reversible events, why do many existing languages allow for variation? All in all, the data indicates that the way in which word order conventions are formed in real life is probably more complex than we have sketched so far, an observation that is central in Levshina et al. (2021), who propose that linguists need to approach word order as a gradient phenomenon rather than labeling languages as belonging to fixed categories. Another option is that the SOV preference for non-reversible events and the SVO preference for reversible events are in fact stronger than the more general bias to use word order completely consistently. Future work on word order in learning and interaction can potentially shed more light on this (see Motamedi et al., 2021b for a novel methodology that explores this for intensional vs. extensional events).

Our experiments have increased our understanding of the word order preferences for different events. By acknowledging that reversibility is a composite phenomenon, and by showing that this is reflected in word order preferences in silent gesture, we have presented additional evidence in favor of the noisy channel account of word order. By extending the silent gesture methodology to an interactive setup, we have made it possible to investigate participants’ preferences regarding word order regularity in more detail. Together, our experiments give the most detailed picture yet of the ordering preferences for conveying who does what to whom.

## Data Availability Statement

The datasets presented in this study can be found in online repositories. The names of the repository/repositories and accession number(s) can be found below: https://osf.io/2pr48/ and https://osf.io/fk6yq/.

## Ethics Statement

The studies involving human participants were reviewed and approved by the PPLS Ethics Committee University of Edinburgh. The patients/participants provided their written informed consent to participate in this study.

## Author Contributions

MS, DN, and SK designed the experiments. DN ran the experiments and prepared the data. MS did data analysis and wrote the manuscript. All authors contributed to the article and approved the submitted version.

## Conflict of Interest

The authors declare that the research was conducted in the absence of any commercial or financial relationships that could be construed as a potential conflict of interest.

## Publisher’s Note

All claims expressed in this article are solely those of the authors and do not necessarily represent those of their affiliated organizations, or those of the publisher, the editors and the reviewers. Any product that may be evaluated in this article, or claim that may be made by its manufacturer, is not guaranteed or endorsed by the publisher.

## Footnotes

1 It should be noted that silent gesture never takes place in the absence of a conventional language, since all participants are language users. We will discuss the potential influence of this in the general discussion.

2 In the number of participants, we deviated slightly from the preregistration: we intended to run 20 participants in each condition, but due to the pandemic and the forced closure of our labs, we were only able to test 15 in each condition.

3 We used R (version 4.0.2) (R Core Team, 2020) with packages lme4 (version 1.1-23) and lmerTest (version 3.1-2). All data and analysis code can be found on https://osf.io/2pr48/.

4 Eight further participants were recruited, but their data were not included for analysis, either because the experiment software crashed during the experiment (in three cases) or they partook in an improvised single participant setting because the scheduled partner participant did not show.

5 All data and analysis code can be found on https://osf.io/fk6yq/. Following the suggestion of a reviewer, we ran a more complex model than the one described in the pre-registration. However, we did run the preregistered model too. This model only had round as a predictor, and did not have a log transformation of round (it was linear instead). It confirmed a main effect of round on entropy. For the full model details, and comparisons in the analysis code on OSF.
